# Correction to: A Natural Variation in *PLEIOTROPIC DEVELOPMENTAL DEFECTS* Uncovers a Crucial Role for Chloroplast tRNA Modification in Translation and Plant Development

**DOI:** 10.1093/plcell/koac013

**Published:** 2022-02-15

**Authors:** Hui Liu, Ding Ren, Ling Jiang, Xiaojing Li, Yuan Yao, Limin Mi, Wanli Chen, Aowei Mo, Ning Jiang, Jinshui Yang, Peng Chen, Hong Ma, Xiaojin Luo, Pingli Lu

www.plantcell.org/cgi/doi/10.1105/tpc.19.00660; Vol. 32, pp. 2345–2366

In the originally published version of this manuscript, errors were introduced at the proof stage with respect to superscript symbols corresponding to authors contributing equally and authors for correspondence.

The first two authors, Hui Liu and Ding Ren, contributed equally to this manuscript. The symbols following their names originally appeared as follows:

Hui Liu,^a^ Ding Ren,^a,b,1^

They now appear as follows:

Hui Liu,^a,‡^ Ding Ren,^a,b,‡^

Xiaojin Luo and Pingli Lu are the authors for correspondence. The symbols following their names originally appeared as follows:

Xiaojin Luo,^a,e,1^ and Pingli Lu^a,b,1^

They now appear as follows:

Xiaojin Luo^a,e,^* and Pingli Lu^a,b,^*

Additionally, the superscript appearing before the addresses for correspondence has been changed from ^1^ to *. The superscript appearing before the line “These authors contributed equally to this work” has been changed from ^1^ to ^‡^.

We apologize to the authors for the errors above.

